# Interface between biomedical and traditional systems of treatment and care among HIV positive fisher folk in two fishing communities on Lake Victoria, Uganda

**DOI:** 10.4314/ahs.v21i3.11

**Published:** 2021-09

**Authors:** Christopher Tumwine, Peter Aggleton, Stephen Bell

**Affiliations:** 1 School of Medicine, Kabale University, Kabale, Uganda; 2 Centre for Social Research in Health, UNSW Sydney, Sydney, Australia; 3 School of Sociology, The Australian National University, Canberra, Australia; 4 Centre for Gender and Global Health, UCL, London, UK; 5 UQ Poche Centre for Indigenous Health, The University of Queensland, Brisbane, Australia; 6 School of Public Health, The University of Queensland, Brisbane, Australia

**Keywords:** HIV care, fisherfolk, HIV, Uganda, traditional healers, anti-retroviral therapy

## Abstract

**Background:**

Fisherfolk have been identified as a key population in the HIV response in Uganda due to high HIV prevalence and low engagement in HIV services. While studies have examined lifestyles and risk, much remains to be understood about help and health seeking experiences, including the combined use of biomedical and traditional health care.

**Objective:**

To examine the use of biomedical and traditional health care in two fishing communities around Lake Victoria in Uganda.

**Methods:**

Exploratory, in-depth qualitative study involving semi-structured interviews with 42 HIV positive fisherfolk.

**Results:**

Prior to HIV diagnosis, participants who described becoming ill sought different forms of help including biomedical treatment prescribed by health workers or self-prescribed; biomedical and herbal medicines together; herbal medicines only; or no form of treatment. Following HIV diagnosis, the majority of participants used ART exclusively, while a smaller number used both ART and traditional care strategies, or reported times when they used alternative therapies instead of ART. Prior to HIV diagnosis, fisherfolk's health care seeking practices inhibited engagement with HIV testing and access to biomedical HIV treatment and care. After HIV diagnosis, most resorted only to using ART.

**Conclusion:**

Study findings provide insight into how fisherfolk's use of biomedical and traditional care prior to diagnosis influences subsequent engagement with HIV treatment. Efforts are needed to reach fisherfolk through everyday health seeking networks to ensure HIV is diagnosed and treated as early as possible.

## Introduction

International studies on the effectiveness of biomedical treatment-based approaches to HIV prevention^1^, ^2^, updated international guidelines^3^, ^4^ and UNAIDS' 90-90-90 targets^5^, ^6^ have led to an expansion of universal test and treat programmes^7^. With a focus on clinical pathways based on the HIV care continuum framework^8^, these aim to move ‘key populations’ quickly through diagnosis and treatment initiation to engagement in ongoing care to ensure viral suppression^7^. In Uganda, the engagement of fisherfolk – identified as a key population due to an estimated HIV prevalence of 22–42% 9, 10 – in HIV care services has been prioritised in national HIV guidelines^11^.

In-depth qualitative research can shed light on influences on fisherfolk's access to HIV testing, treatment and care and treatment adherence. Factors inhibiting fisherfolk's engagement with HIV care services along the HIV care continuum include mobility and transport challenges associated with fishing livelihoods^12^–^14^, masculine occupational identities^15^, alcohol use 13,16 and personal health-related decision making 17. Interpersonal relations and social support can enable fisherfolk's access to HIV care and treatment 18–20.

The concurrent use of biomedical and traditional systems of health care for HIV is widely documented in Uganda^21^ and other sub-Saharan settings 22, 23. In such contexts, ‘biomedical care’ may be defined as HIV care implemented in accordance with national HIV policies, involving the use of anti-retroviral therapy (ART) provided by biomedically trained health workers in clinical settings^22^. Alternatively, ‘traditional’ health care is informed by beliefs, values and customs shared between generations and involves the use of herbal medicines and other forms of treatment including various forms of spiritual healing^22^.

In Uganda, research has documented concomitant use of herbal medicine and ART by HIV positive people 21, 24, 25. Studies report delays in treatment initiation 17,13 due to herbal medicine use. Yet to date fisherfolk research has largely focused primarily on ART and biomedical health services 12, 15, 16, 18, with little attention paid to the interplay between biomedical and traditional systems of care at different stages along the HIV care continuum, and particularly prior to HIV testing among people experiencing symptoms. National HIV responses in Uganda would benefit from improved understandings of why fisherfolk use biomedical and traditional care systems, and how usage changes with time and affects fisherfolk's use of HIV testing and ART adherence. Such data is as yet unavailable in this national setting.

Drawing on in-depth interviews with HIV-positive fisherfolk in south-central Uganda, we contribute to these knowledge gaps. The aim of this paper is to examine fisherfolks' perspectives and experiences of the use of biomedical and traditional health care, prior to and after HIV diagnosis, and the impact that these practices have on HIV diagnosis and treatment initiation.

## Methods

### Study design and setting

This qualitative study was conducted in one lakeshore community and one island community on Lake Victoria in south-central Uganda during 2016–2017. At the time of the study, fisherfolk on the lakeshore had access to HIV testing, treatment and care services through three public sector health facilities and two health facilities run by non-governmental organisations. On the island, HIV testing, treatment and care services were offered through one public sector health facility and one health facility run by a non-governmental organisation. HIV treatment was largely provided free of charge, with transport costs for appointments ranging between UGX 2000-150,000 (GBP 0.43–32.62). Approximately equal numbers of traditional healers were living in lakeshore and island communities.

### Sample and recruitment

Fisherfolk were recruited purposively through a community clinic run by The AIDS Support Organisation (TASO) in Entebbe Municipality on the shores of Lake Victoria and a government clinic (Kalangala Health Centre IV) on Bugala Island in Lake Victoria. Expert clients, understood as volunteer experienced HIV positive persons who are trained to support other HIV positive persons at the clinic and in community settings, introduced the study to peers at each clinic and in local communities. Prospective participants were invited to participate and referred to the first author to complete the consent process. Initial contact with potential participants was made by expert clients to introduce the study; the aim of this arrangement was to ensure that prospective participants did not feel coerced to participate in the study.

Inclusion criteria were: 18 years and older; HIV positive; and categorised as a ‘fisherperson’, defined as living (within a radius of 2km) or working at a fish landing site or fishing community; or a person whose livelihood was directly or indirectly derived from fishing. Recruitment of study participants continued until such a point where it was deemed that data saturation had been achieved. Data saturation generally refers to the idea that recruitment of study participants ends at the point when no new information is being generated by further interviews^26^.

### Data collection

All interviews followed a pre-tested semi-structured discussion guide to elicit detailed narratives of participants' experiences accessing and using HIV testing, treatment and care. A semi-structured interview is a dialogue between a researcher and participant, guided by a flexible interview protocol which is supplemented by follow-up questions and probes, enabling the researcher to follow up on a participant's responses while covering themes relating to the study^27^. The guide explored a range of themes including health and help seeking experiences in the two years prior to HIV diagnosis and in the period that followed this. This analysis focuses on discussions pertaining to participants' use of biomedical and traditional health care services. Interviews were conducted in participants' first language (Luganda or Runyankole) by the first author and a research assistant, in audio-private locations in clinic or community locations. Interviews lasted 42–148 minutes and were audio-recorded, transcribed and translated into English by the first author and a research assistant who are both fluent in Luganda and Runyankole.

### Data analysis

All interview transcripts were anonymised and then uploaded into ATLAS.ti software to facilitate coding. Data were coded in accordance with the principles of thematic analysis using deductive and inductive techniques^28^. Deductive techniques informed by the HIV care continuum framework^8^ were used to examine the health seeking experiences of fisherfolk before and after HIV diagnosis. Data in these two periods (i.e. before and after HIV diagnosis) were subsequently analysed inductively to develop codes identifying specific issues relating to participants' biomedical and traditional care practices.

### Ethics

Ethical approval was obtained from UNSW Sydney (HC 16545) and TASO Uganda (TASO-REC35/16-UG-REC-009). The study protocol was reviewed and registered by the Uganda National Council for Science and Technology (SS 4145). Pseudonyms are used throughout this paper to ensure participant anonymity.

### Researcher reflexivity

The interviews were conducted by the first author and a research assistant. The first author is a Ugandan man, fluent in languages spoken in the fishing communities involved in this study. He has worked extensively with HIV-positive people before and therefore has ‘insider’ understandings of many of the issues that arose during the research. Yet he was also an ‘outsider’ given that he was not a member of the fishing communities. This outsider status meant that he had a lot to learn about fisherfolk's ways of life, lifestyles and terminology used to describe their lived experiences. The research assistant was also a local man with prior experience conducting research with HIV-positive persons, fluent in Luganda and Runyankole, and a recent graduate in social sciences. Due to these shared experiences, it was possible to build good rapport with most participants despite the vast differences in life experience, especially by gender – and elicit in-depth insights into fisherfolk's lives. The depth and quality of the life stories gathered during interviews ensured fisherfolk's own experiences and perspectives are central to the analysis provided in this paper.

## Results

Interviews were conducted with 42 HIV positive fisherfolk. Detailed socio-demographic characteristics of the participants are presented in Table 1. Reported health seeking practices from biomedical and traditional health care systems varied before and after HIV diagnosis.

**Table 1:** Characteristics of study participants

Characteristics	Number
Gender - Male - Female	24 18
Age - 18–30 - 31–40 - 41–50 - 51+	4 19 13 6
Location of residence - Island - Lake shore	30 12
Marital status - Single - Married - Divorced/Separated - Widowed	0 24 13 5
HIV diagnosis year - 2000–2010 - 2010+	21 21
ART status - On ART - Not on ART	39 3
Fisherfolk role* - fish catcher (boat crew) - farmer - fish processor - casual labourer - construction worker - Agricultural products trader - Lodge/bar/restaurant - fish trader - Others	16 15 4 4 2 4 3 6 2

* Several participants performed more than one rol

### Fisherfolk's health care seeking practices prior to HIV diagnosis

Of the 42 study participants, 27 reported feeling unwell prior to HIV diagnosis. To deal with symptoms including fever, diarrhoea, skin problems, headache and coughing, 18 used only biomedical treatments, one used only herbal medicines, six used a combination of biomedical and herbal medicines, and two participants did not seek care.

*Use of biomedical treatment*

Participants used biomedical treatment because it was more readily available or because they perceived such treatment to be more effective. Medicines were either self-prescribed, where, ‘when some become ill, they go to a pharmacy and buy drugs to treat themselves’ (Levi, 49 years old) or were prescribed by biomedical care providers such as general practitioners.

*When most people in this community become sick, they come to this [government] health centre for treatment... some opt to go to private clinics because they are able to get medicines when they pay money at the private facilities*. (Mary, 36 years old)

When seeking support from health services, some interviewees described long periods of time before the link was made to possible HIV infection. Narratives illustrate how interactions with health service providers about worrying symptoms did not always lead quickly to HIV testing and diagnosis.

*Before I came to know that I was HIV positive... I would feel quite hot, and sweat a lot, get a lot of headache for quite long... I went to the clinic and received treatment, but the illness was not going away. I even went to Jinja hospital and was treated for diarrhoea, but the illness still did not go away*. (Umar, 61 years old)

*I never took any herbal medicines before I came here [at the HIV clinic]. I got only injections and treatment for the chest pain. And that was largely because the small clinics I went to do not have laboratories to talk of, so they use some guesswork to treat patients. I got treatment for chest pain and nausea.* (Zainabu, 40 years old)

*Blending traditional and biomedical care solutions*

Participants described a range of herbal medicines that were used to treat illness, including ‘omululuza’ (bitter leaf) and ‘ekigaji’ (aloe vera) (Audrey, 40 years old), ‘herbal medicines to drink’ (Edwin, 48 years old), and ‘wood ash to treat skin conditions associated with herpes zoster’ (Simon, 39 years old). Some medicines were provided by traditional healers, while other fisherfolk collected and prepared medicines themselves according to teaching from parents and grandparents.

*I got the plants I used to process herbal medicines from the bush myself and prepared them and took the medicine. When I was growing up, I would see my parents preparing these medicines for us to take*. (Audrey, 40 years old)

Some participants used biomedical and herbal medicines to treat illness, informed by the hope that at least one strategy would cure symptoms.

*I do not get better even when I take modern antimalaria pills. But when I take herbal medicine, I get better much quickly... I may take malaria pills in the morning and take herbal medicine in the evening. I do not mind which of the two has healed me. What I would be in need of is to get better.* (Paul, 36 years old)

Others started with herbal medicines before trying biomedical treatments when symptoms did not improve.

*When I was not feeling fine, I prepared herbal medicine and took it for two months but never got better... After that, I took modern medicines to treat various illnesses that I was experiencing until I realised I was using all the money I was earning and not getting better*. (Levi, 49 years old)

Irrespective of seeking care in biomedical or traditional systems, participants reported little or no improvement to illnesses they experienced. Some participants spent a long time seeking care for different illnesses from traditional and/or biomedical health care providers before being offered an HIV test. They only overcame health probems after HIV diagnosis and initiating anti-retroviral therapy.

*After learning that I was HIV positive, I never used herbal medicines again. After testing HIV positive, I started taking Septrin and later on ARVs when my CD4 count lowered. And after I started taking HIV medications, I do not fall sick quite frequently*. (Audrey, 40 years old)

*Fisherfolk's health care seeking practices following HIV diagnosis* After HIV diagnosis, 26 participants used ART exclusively, 13 used ART and traditional care strategies, while 3 reported periods when they used traditional care strategies instead of ART.

*Use of ART only*

Many participants stopped using herbal medicines as soon as they learned they were HIV positive and started taking ART. This was because they perceived the latter to be more efficacious in the treatment of HIV compared to herbal medicines. Adherence to ART was encouraged because interviewees described ongoing experiences of good health since starting treatment as well as the ability to work and provide for their families.

*My ARVs are my life. ...Many HIV positive people in our home village died before these ARVs became available. But for me, because I am taking these drugs, you may not be able to tell I am sick and HIV positive, because I am healthy. I am able to do my work as usual without any hindrances, and I am able to earn income and pay for my children's school fees.* (Sarah, 46 years old)

Other participants reported learning that herbal medicines inhibited the effectiveness of ART.

*We [I and my wife] used herbal medicines at some point. It was sold to us by someone who indicated it helps to manage HIV among HIV positive persons. Then afterwards I heard that when you take herbal medicines, they obstruct the effect of modern medicines*. (Denis, 53 years)

Participants also explained that the herbal medicines supplied by traditional healers were costly compared to ART which was provided largely free of charge at HIV clinics, which was another reason for using ART only.

*There were a group of people who have ever approached me marketing their traditional medicine as good in treating HIV. But when I continued to discuss with them I realised that their medicine was quite expensive and I could not afford it. And the good thing, I receive free treatment from here, so, I chose to remain here, receiving ART.* (Edmund, 37 years old)

*Combining ART and herbal medicines*

While using ART as prescribed, 13 participants also used herbal medicines occasionally to treat HIV-related opportunistic infections and ART side effects.

*I took this [herbal] medicine after I tested HIV positive. I was taking only Septrin at the time I used this herbal medicine. I took about five bottles and my wife also took about five bottles of this herbal medicine... I had been told that it will restore my strength and will help to reduce the HIV virus in my body.* (Denis, 53 years old)

Other people reported the use of herbal medicines to treat illnesses perceived as unrelated to HIV.

*I sometimes take traditional medicine when I am not feeling well... When I get problems such as ulcers, tooth aches, malaria, I use traditional medicine to treat them.* (Michael, 43 years old)

*Substituting ART for herbal medicine and other forms of treatment*

Three participants described not initiating ART, at least for a short period of time following HIV diagnosis, due to their preference for herbs or other forms of treatment. One used herbal medicines, another sought out and ate ‘nutritious food’, but the third refused to believe his HIV diagnosis and continued using herbal and other biomedical treatment instead of ART. Each reported eventually abandoning these strategies and, at the time of interview, were all using ART exclusively.

*I was still strong by the time I tested HIV positive... I was able to eat good food ... so I never had any reason to take ARVs... when the HIV symptoms started becoming apparent, I realised that eating only nutritious food was not enough to sustain me... I realised if I do not start taking ARVs, then HIV was heading to ‘eat up’ my life now*. (Moses, 40 years old)

## Discussion

Consistent with findings from other research with HIV positive people in Uganda^13^, ^17^, ^21^, ^24^, ^25^ and elsewhere in Sub-Saharan Africa^22^, ^23^, findings from this study confirm that fisherfolk access traditional and biomedical health care systems for care prior to and after HIV diagnosis, and alongside ART. In contrast to other published research, our analytic focus on fisherfolk experience prior to and after HIV diagnosis offers a diachronic perspective on these health seeking practices, pointing to some of the challenges faced when transitioning into the formal HIV care system. In particular, it documents how the use of traditional health care was more prominent prior to HIV diagnosis than afterwards, and how the use of herbal medicines inhibited engagement with HIV testing for some participants. Following diagnosis, however, respondents reported little impact of traditional care on initiation of, or adherence to, ART.

HIV testing is the entry point to the biomedical health care system and enables people with HIV to be diagnosed, initiate early treatment and live healthy lives^3^–^6^. In this study, a lack of symptoms prior to an HIV positive diagnosis inhibited HIV testing among fisherfolk. For participants with symptoms, engagement in early HIV testing was inhibited by the interplay between treating illness within traditional and biomedical care systems, as well as by seeking care from non-specialist HIV health workers who did not make the link between symptoms and undiagnosed HIV infection.

Existing qualitative studies with HIV positive populations in Uganda have suggested that the the continued use of traditional medicines can inhibit treatment initiation^17^ or interrupt treatment adherence^13^. In contrast, the vast majority of fisherfolk participants in this study reported prioritising access to ART after an HIV-positive diagnosis, most of these stopped using all herbal medicines. The long-term use of biomedical HIV care and treatment was reported due to experiences of good health while taking ART, the low cost of ART drugs and the perception that herbal medicines might inhibit the effectiveness of ART.

Through its novel examination of the interface between fisherfolk's use of biomedical and traditional health systems for HIV care, study findings extend understanding of the diverse influences on HIV prevention and treatment reported in other studies, include socio-ecological issues^12^, ^13^, ^15^, ^17^–^19^. To date, health systems research with fisherfolk has largely documented experiences of biomedical health services only^12^, ^15^, ^18^. The few studies on concurrent use of herbal medicines and biomedical treatments in Uganda have largely involved more general HIV-positive populations with a focus on participants' experience following HIV diagnosis alone^13^, ^17^, ^21^, ^24^, ^25^. Filling these research gaps, this study offers insight into how fisherfolk's use of traditional and biomedical care prior to diagnosis may influence engagement with HIV treatment subsequently, with a view to enhancing engagement in early HIV testing (irrespective of symptoms) and detection, and early treatment initiation for those who need it.

## Limitations

This study has limitations. The sample size was small, drawn from two clinics and findings may not be generalisable beyond the study settings. Triangulation of findings between island and mainland residents and deep rapport with study participants established by the time spent by the lead author in the study locations, lengthy interviews, and the conduct of research in local languages enhanced internal and ecological validity, and the trustworthiness of findings^29^, ^30^. It will be useful for quantitative research to explore and validate the patterns we have observed in larger more representative community samples.

## Implications for policy and practice

Findings have important implications for HIV testing, treatment and care. Significant effort is required to reach fisherfolk with HIV testing to ensure HIV is diagnosed early. Within fishing communities, this could mean the establishment of referral systems via local pharmacies and drug stores, traditional healers and religious institutions, with a focus on symptoms that might indicate the need for regular HIV testing. Engaging traditional healers in sensitisation and education to encourage clients to seek a biomedical diagnosis would supplement these actions. The involvement of fisherfolk who are expert clients and who have benefitted from ART leading HIV prevention and treatment communication in community settings would also be beneficial, supporting renewed emphasis on the importance of community-led responses to HIV prevention^31^.

## Conclusion

Findings illustrate fisherfolk's health seeking practices from traditional and biomedical health care systems as they seek HIV care. Interplay between these systems inhibited engagement with HIV testing but did not inhibit treatment initiation or treatment adherence following an HIV diagnosis in this sample. Efforts are needed to reach fisherfolk with HIV testing made available through everyday health seeking networks to ensure that HIV is diagnosed and treated as early as possible.
